# Regulation of Vitamin C Homeostasis during Deficiency

**DOI:** 10.3390/nu5082860

**Published:** 2013-07-25

**Authors:** Maiken Lindblad, Pernille Tveden-Nyborg, Jens Lykkesfeldt

**Affiliations:** Section of Experimental Animal Models, Department of Veterinary Disease Biology, Faculty of Health and Medical Sciences, University of Copenhagen, Ridebanevej 9, Frederiksberg C 1870, Denmark; E-Mails: mali@sund.ku.dk (M.L.); ptn@sund.ku.dk (P.T.-N.)

**Keywords:** vitamin C transport, sodium-dependent vitamin C transporters, SVCT1 and SVCT2, glucose transporters (GLUTs), regulation of transport

## Abstract

Large cross-sectional population studies confirm that vitamin C deficiency is common in humans, affecting 5%–10% of adults in the industrialized world. Moreover, significant associations between poor vitamin C status and increased morbidity and mortality have consistently been observed. However, the absorption, distribution and elimination kinetics of vitamin C *in vivo* are highly complex, due to dose-dependent non-linearity, and the specific regulatory mechanisms are not fully understood. Particularly, little is known about how adaptive mechanisms during states of deficiency affect the overall regulation of vitamin C transport in the body. This review discusses mechanisms of vitamin C transport and potential means of regulation with special emphasis on capacity and functional properties, such as differences in the *K*_m_ of vitamin C transporters in different target tissues, in some instances demonstrating a tissue-specific distribution.

## 1. Introduction

Several reports have pointed towards a putative link between vitamin C (vitC) deficiency and lifestyle-associated disease [1,2,3,4,5,6]. Moreover, vitC deficiency has also been associated with impaired brain development and reduced hippocampal function in experimental animal studies [7,8,9,10]. Scurvy is the terminal outcome of a prolonged period of severe vitC deficiency (a plasma concentration <11 μM [11]) and is rarely encountered, as it can be prevented with as little as 10 mg vitC/day. In contrast, marginal vitamin deficiency or hypovitaminosis C, defined as a plasma concentration below 23 μM [11], has been estimated to affect as much as 10% of adults in the industrialized world [12,13,14], with subgroups, such as smokers and families with a low socio-economic status, displaying an even higher prevalence [15,16,17,18,19]. Adverse effects of marginal vitC deficiency could, therefore, potentially affect a substantial amount of people who, unknowingly and devoid of known clinical symptoms, are at risk of experiencing negative long-term effects of vitC deficiency, including increased mortality associated with a variety of disease complexes [20].

The pharmacokinetics of vitC, *i.e.*, the absorption, distribution, metabolism and elimination, is quite complex and involves several different active and passive transport mechanisms, as well as intracellular reduction permitting the recycling of vitC within specific tissues [18,21]. Furthermore, vitC is differentially distributed between tissues, with brain and neurons, in particular, upholding a much higher concentration than most other organs [22,23,24]. However, whereas vitC homeostasis has been studied in detail during sufficiency [25,26,27,28], less is known about the potential adaptive mechanisms during deficiency. Thus, for example, studies of vitC transport activity during deficiency have not provided evidence supporting a direct relationship between local tissue-concentration and the expression of vitC specific transporters [29,30,31], prompting suggestions of possible alternative transport mechanisms [29]. The present review elaborates on the mechanisms currently known to be involved in the regulation of vitC transport and the potential effects during states of deficiency.

## 2. Vitamin C Transport

**Figure 1:**
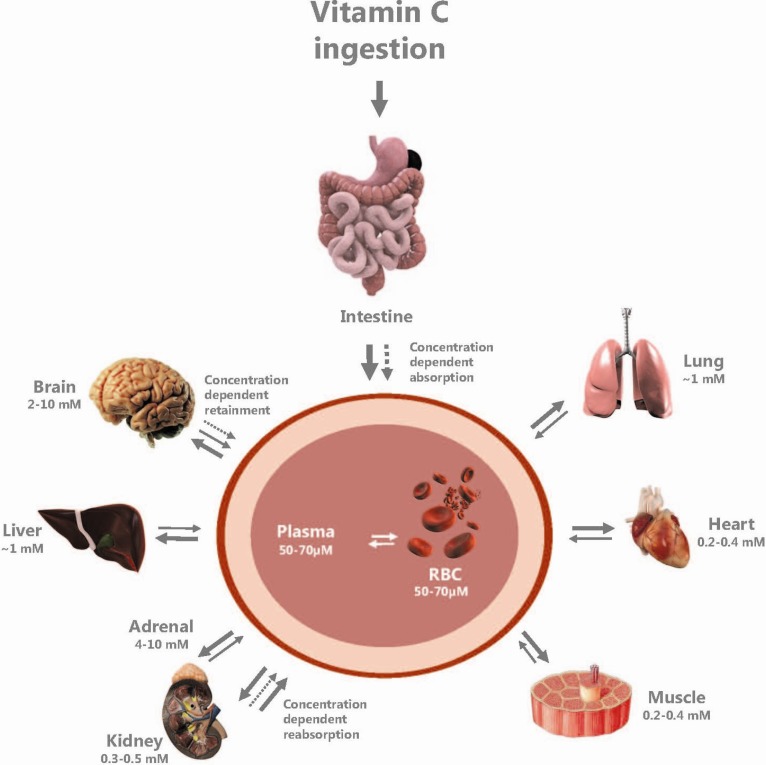
Distribution of vitamin C in the body. Vitamin C is ingested, absorbed from the intestinal lumen and transported to various peripheral organs with the blood. Finally, vitamin C is excreted in the renal glomeruli and reabsorbed through the tubular systems. Tissue concentrations are dependent on all of these processes.

Most mammals synthesize vitC in the liver by enzymatic conversion of glucose; however, a few species, including humans, guinea pigs and bats, lack a functional l-gulono-lactone oxidase enzyme catalyzing the final step in the biosynthesis and, therefore, rely completely on a dietary supply of vitC [32,33,34]. After consumption, vitC is absorbed from the intestinal lumen and released into the bloodstream. The dose-to-plasma concentration relationship is reflected in a saturation curve, attaining an initial steep and non-linear course, until steady-state is reached, defining plasma saturation at around 70 μM in humans [25]. At doses above saturation, urinary excretion is increased and oral bioavailability decreased, thereby sustaining steady-state equilibrium [25]. In the gastro-intestinal tract, the ionized form of vitC, ascorbate (ASC), and its oxidized counterpart, dehydroascorbic acid (DHA), are absorbed through different transporter systems with an increased affinity for ASC (*K*_m_ of 0.2 mM) compared to DHA (*K*_m_ 0.8 mM) and following a Michaelis-Menten absorption rate, where saturation reflects increases in substrate concentration [21,35]. The distribution from plasma to tissues is differentially regulated, and organs and tissues vary considerably in vitC content, as depicted in Figure 1. Within the body, the brain has a uniquely high vitC level [22,36] and is able to maintain a superior concentration relative to most other organs during periods of vitC deficiency [37,38,39], placing the brain as an organ of particular interest when assessing the effects of vitC deficiency.

The tight regulation of vitC homeostasis is primarily controlled by four regulatory systems: (i) intestinal uptake (bioavailability); (ii) tissue accumulation and distribution; (iii) rate of utilization and recycling; and (iv) renal excretion and reabsorption [26] (Figure 1). This is achieved by different mechanisms, including passive diffusion, facilitated diffusion, active transport and recycling [40].

### 2.1. Passive Diffusion

In solution at physiological pH, vitC predominantly attains its ionized form, ASC. Because of a relatively low hydrophobicity, ASC and DHA do not easily cross biological membranes and primarily rely on the interaction with transporter molecules anchored in the cell membrane [41,42]. However, both ASC and DHA can, to a small extent, move by passive diffusion, and DHA diffuses through cellular membranes more willingly than ASC [43]. It has also been suggested that during intestinal uptake and in renal reabsorption, ASC leaves the epithelial cells from the basolateral membranes down a concentration gradient by passive diffusion [44]. Moreover, being a week acid with a pKa of 4.2 [21], the theoretical equilibrium between blood and tissue favors plasma with a ratio of 2.5 to one. Regardless, the fraction by which passive diffusion contributes to the overall regulation of vitC homeostasis at physiological conditions is believed to be of minor importance [40].

### 2.2. Facilitated Diffusion

Transport of DHA occurs by facilitated diffusion, enabling transport along a concentration gradient. This gradient is maintained as DHA is reduced to ASC immediately after crossing the membrane [21,45,46,47]. DHA, but not ASC [48,49], is transported by facilitated diffusion through four of the 14 glucose transporters (GLUT 1–4), although with varying affinities and efficiencies [47,48,49,50]. In general, these transporters have 12 transmembrane domains and are approximately 500 amino acids-long [51]. The GLUT-transport of DHA is competitively inhibited by glucose, e.g., excess glucose in plasma or intestine will block the receptor-binding site and, subsequently, decrease GLUT-facilitated DHA transport [21,47,48]. This association is also seen for DHA absorption to some specific cell types, whereas in others, glucose has less or even no significant effect on DHA absorption (*i.e.*, the luminal surface of absorptive gut epithelium and in tubular cells in the kidney) [21,35,47,52,53].

The distribution and transport properties vary among the different GLUTs. Thus, GLUT1 is expressed in a broad variety of cells throughout the body [48,51]; GLUT2 is primarily expressed in liver, spleen and the basolateral membrane of intestinal and renal epithelial cells [50,51]; GLUT3 is found particularly in the brain and in neurons [48,51] and GLUT4 in skeletal and cardiac muscle cells, as well as in adipose tissues [47,51].

### 2.3. Recycling

Following uptake across the intestinal epithelium, vitC is released into the bloodstream as ASC (>95% of vitC in human plasma is in the form of ASC [52,54]). Here, ASC is easily oxidized and the produced DHA rapidly taken up through GLUT1 transporters on the erythrocytes [55,56,57]. May and co-workers have demonstrated that DHA is rapidly recycled in erythrocytes predominantly via glutathione-dependent DHA reductases and with small contributions from reduced nicotinamide adenine dinucleotide phosphate (NADPH)-dependent DHA reductases, such as thioredoxin reductase [58,59,60,61,62,63,64,65,66]. The resulting ASC may subsequently be released to the bloodstream [65]. During oxidizing conditions in the extracellular space, the generated DHA is absorbed by the surrounding cells and immediately reduced [45,55]. This reduction of DHA to ASC is either by chemical reduction by glutathione (GSH) [67] or by enzymatic reduction by glutaredoxin [68,69,70], protein disulfide isomerase (PDI) [69,70] or 3-α-hydroxysteroid dehydrogenase [71]. The mechanism by which DHA is reduced to ASC is a continuous process within the cellular cytoplasm, sustaining adequate levels of ASC to quench free radicals, such as superoxide, and/or reduce other antioxidants, such as vitamin E, maintaining redox homeostasis [56,72,73]. The rapid intracellular recycling of DHA also drives the efficient facilitated diffusion of DHA through the GLUTs, as the intracellular concentration is kept sufficiently low to allow DHA to move along a concentration gradient.

### 2.4. Active Transport of ASC

The existence of specific vitC transporters were proposed long before the actual transporters could be identified [42,74,75,76,77,78,79,80,81,82]. The transport was found to be concentration-, energy-, temperature- and sodium-dependent, satiable and mediated by two different components [42,74,75,76,77,78,79,80,81,82]. Two specific transporters were defined by Tsukaguchi and co-workers as sodium-dependent vitamin C transporter (SVCT) 1 and SVCT2 [83]. These enable the active transport of ASC against a concentration gradient, allowing an accumulation in cells of concentrations more than 50-fold that of extracellular fluids [76,77]. The sodium-dependency of the transport has been shown to have a stoichiometry of two Na^+^-ions to one ASC anion [84,85,86], demonstrating the transport as a secondary active transport, with the sodium gradient driving the transport of ASC, which, in turn, is maintained by the sodium/potassium-ATPase.

In humans, SVCT1 is a 598 amino acid protein, while SVCT2 measures 650 amino acids [87]. The proteins are encoded by the genes, *SLC23A1* (located at chromosome 5q31.2–31.3) and *SLC23A2* (at 20p12.2–12.3), respectively [88,89,90,91]. The transporters share 65% identity [83] and are differentially distributed within the body. The SVCT1 is primarily expressed in epithelial cells [90,92,93]. It has a relatively high *K*_m_ of 65–252 μM [52,87,94,95,96,97] and a *V*_max_ of approximately 15 pmol/min/cell [87,90], establishing SVCT1 as a low affinity/high capacity transporter and corresponding well to its function believed to be maintaining whole-body homeostasis [52,87,96,97,98]. The SVCT2 is expressed in various organ systems [83]. SVCT2 has high affinity (*K*_m_-values of 8-69 μM [52,87,94,95,96,97]), but low capacity (approximately 1 pmol/min/cell [87,90]) for vitC transport [52,87,96,98], enabling cells of peripheral organs to take up ASC from the extracellular fluid [40,97]. The SVCT2 transporter is believed to be the primary transporter of ASC to the brain, enabling this organ to obtain and preserve a strikingly high vitC concentration, even during states of (severe) deficiency [37,38,39]. The distribution of the two transporters taken together with their kinetic properties suggests a distinct polarity of expression with SVCT1 expressed on the apical side of the epithelial cell membrane, while SVCT2 is suggested to be located in the basolateral membrane [52]. This has been confirmed by *in vitro* studies displaying apical SVCT1 expression and SVCT2 expression in the basolateral membrane of enterocytes and renal tubule cells [99,100], supporting the distinctive roles of the two transporters in regulating overall vitC concentration; SVCT1 adhering to luminal surfaces in the intestinal tract and kidney and SVCT2 being associated with the further distribution and/or re-uptake of ASC [97,101]. Findings in Caco-2 cells, an *in vitro* model of human enterocytes, suggest that the basolateral SVCT2 primarily transports ASC into the enterocytes from the blood stream, while not contributing to the absorption of dietary vitC as such [99].

Importantly, recent knowledge of human genetic variation in the SVCTs and its impact on vitC homeostasis has complicated the interpretation of the existing clinical literature (for a comprehensive review, see [102]. Using pooled human genotype data, Corpe and coworkers calculated average population prevalences of *SLC23A1* polymorphisms, and following an extensive experimental *in vivo* study in mice, they modeled dose *vs.* plasma concentrations in an attempt to predict human steady-state levels as a function of known allelic genotypes [103]. Their results surprisingly suggest that, e.g., the A772G allele results in a vitC deficient phenotype regardless of the vitC intake (up to 2500 mg/day). Genetic variation is also well known in *SLC23A2*, with about 2200 identified single-nucleotide polymorphisms (SNPs) [102]. However, changes in *SLC23A2* can be expected to primarily influence tissue rather than plasma levels of ASC, and indeed, little is known about their impact on vitC homeostasis in general. Future human studies of vitC homeostasis should preferably include genotyping, as well as assessment of tissue/cellular concentrations through biopsy or leukocyte analysis, in addition to that of plasma.

### 2.5. Vitamin C Distribution

**Figure 2:**
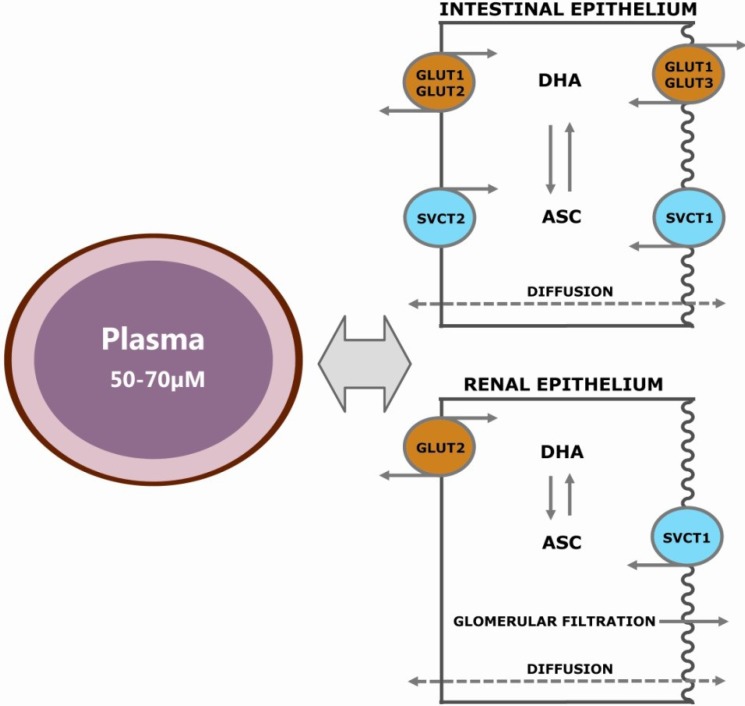
Transport mechanisms between intestines, blood and kidney. Ingested vitC is absorbed across the intestinal epithelium primarily by membrane transporters in the apical brush border membrane, either as ascorbate by sodium-coupled active transport via the sodium-dependent vitamin C transporter (SVCT) 1 transporter or as dehydroascorbic acid (DHA) through facilitated diffusion via glucose transporter (GLUT) 2 or GLUT3 transporters. Once inside the cell, DHA is efficiently converted to ascorbate (ASC) or transported to the blood-stream by GLUT1 and GLUT2 in the basolateral membrane, thereby maintaining a low intracellular concentration and facilitating further DHA uptake. ASC is conveyed to plasma by diffusion, possibly also by facilitated diffusion through volume-sensitive anion channels. SVCT2 located in the basolateral membrane enables re-uptake of ASC from plasma to the intestinal epithelium. In the kidney, ASC is excreted by glomerular filtration to the renal tubule lumen. Reabsorption is primarily achieved by SVCT1 transporters in the apical membrane, although diffusion from the luminal surface may also contribute to the overall uptake. While not confirmed *in vivo*, DHA is presumably re-absorbed in the renal tubule cells; however, the availability of DHA for re-absorption is thought to be negligible, due to the very low DHA concentrations in plasma. As in the intestinal epithelium, ASC can be released to the blood-stream through both passive and facilitated diffusion. GLUT2 transporters are located in the basolateral membrane, enabling transport of DHA to plasma.

The absorption of both ASC and DHA takes place in all segments of the small intestine (duodenum, jejunum and ileum) [35,82,104]. The release of vitC from intestinal and renal epithelial cells after luminal absorption has not yet been conclusively determined, although both passive diffusion and facilitated diffusion through volume-sensitive anion-channels has been suggested [44]. Enterocytes express GLUT1, GLUT2 and GLUT3, although they have distinct cellular locations [105,106,107]. GLUT3 is found on the apical brush-border membrane, while GLUT1 is found on the basolateral membrane, and GLUT2 has been localized on both sides of the membrane. It is possible that vitC enters the blood stream by diffusion through discontinuities in the endothelial wall and circulates in the body with the blood, where it is primarily found as the ASC anion [40] (Figure 2).

The entry of ASC into the brain is hampered by the blood-brain barrier, which is impermeable to ASC and lacks the expression of SVCT2 [108,109,110]. Instead, ASC is thought to enter the cerebrospinal fluid of the brain through SVCT2 transporters in the choroid plexus [110,111]. DHA, on the other hand, readily crosses the barrier, due to the expression of GLUT1 [108,112]. However, due to the limited amounts of circulating DHA compared to the very high ASC concentrations maintained by the brain, this mechanism is generally believed to be of little significance [25,54]. From the extracellular space in the brain, ASC is taken up by neurons through SVCT2 [83,89,113,114]. Inside the neurons, ASC is oxidized to DHA, which is then recycled to ASC or transported out of the neurons as DHA by GLUT3 within the neuronal membrane [113]. The DHA now present in the extracellular space can then be transported to the blood stream by GLUT1 in the blood-brain barrier or taken up by astrocytes expressing GLUT1, recycled back to ASC and, concomitantly, released to the extracellular space by a yet undisclosed mechanism or possibly by diffusion [36,113].

In the renal glomeruli, ASC in the blood is filtered into the urine [115]. However, depending on the vitC status of the individual, a large proportion is reabsorbed along the proximal tubules [116,117]. This reabsorption primarily takes place at the apical side of the epithelial membrane through SVCT1 [40,118,119,120]. It is assumed that DHA is also reabsorbed from the glomerular filtrate, although it has not been confirmed *in vivo*, most likely due to the negligible amounts of DHA commonly found in plasma [55]. In early *in vitro* studies, DHA transport in kidney cells was found to have all the characteristics of the facilitated diffusion through the GLUTs [121,122]. In spite of this, the transport of DHA seems to have an insignificant role in the renal reabsorption, as *Slc23a1^−/−^* mice, unable to reabsorb ASC from the urine, showed an 18-fold increased fractional excretion of ASC that could not be replaced by the uptake of DHA [103].

A recent study in mice further revealed SVCT2 as the functional transporter in the kidney during fetal development, whereas SVCT1 expression rapidly increased in early postnatal life to become the dominant transporter in adult life [123]. The results were supported by findings of SVCT2, but not SVCT1, being expressed in human embryonic kidney cells [123]. These findings demonstrate a developmental shift in both expression and cellular localization of the SVCT transporters, with SVCT2 primarily located at the apical membrane during embryonic development, only to be translocated to the cytoplasm and the basolateral membrane within the first days of postnatal life, following which, the SVCT2 was cleared and replaced by SVCT1 [123]. A shift in SVCT-expression patterns has also been shown for other tissues. In an *in vivo* study in mice, an altered expression of SVCT2 during fetal and postnatal development was found [29]. A marked increase in SVCT2 mRNA and protein occurred from embryonic day E15 to postnatal day P32 in both cortex and cerebellum. In liver, the increase occurred already at P1 for mRNA and P10 for protein. The changes in SVCT2 expression appeared to be directly related to the tissue content of ASC in liver, but inversely related in brain [29].

## 3. Regulation of VitC Transport by Substrate Concentrations

VitC transport can potentially be regulated by the amount of the various transporters or by their state of activity, thus relying on the rate of *de novo* synthesis (and degradation) and on activation of non-functional transporters, putatively also involving cellular translocation. Translational regulation of SVCT2 has been demonstrated *in vitro* in human platelets [124], and both SVCT1 and SVCT2 contain potential sites for glycosylation and phosphorylation as putative targets for post-translational regulation (Table 1). *N*-linked glycosylation sites are located in the second and third extracellular loop, and a single protein kinase A-dependent and several protein kinase C-dependent phosphorylation sites have been described in the amino acid sequence [52]. In humans, enzymatic activity of protein kinase C elicits different responses of SVCT1 and SVCT2. For SVCT1, the cytoplasm-to-membrane translocation is reduced, whereas the phosphorylation elicits a conformational alteration of the SVCT2 [97]. An association between the availability of vitC and an effect on transport mechanisms has been suggested, displaying the characteristics of a substrate-dependent regulation [124,125,126,127,128,129,130].

**Table 1:** Overview of *in vitro* studies regarding the regulation of the transport of vitamin C (vitC) during deficiency.

Cell line	Deficiency regimen	Principal findings
Human intestinal cell line (Caco-2 TC7) [125]	Culture medium was supplemented with ASC at concentrations of 45 μg/mL, 450 μg/mL or 4.5 mg/mL.	Exposure to 4.5 mg/mL ASC significantly reduced the ASC uptake by 50% and expression of SVCT1 mRNA by 77% compared to control conditions.
Primary human platelets [124]	Culture medium’s ASC concentration was reduced to 30% of standard levels.	*V*_max_ increased by 240% in response to the reduction of ASC concentration. A subsequent increase in SVCT2 protein level was reported. An ASC supplement of 500 μM only slightly decreased SVCT2 levels.
Human hepatic cell line (HepG2) [127]	Cells were incubated with 10% fetal bovine serum containing 10 mM ASC (supplemented), 0.7 μM ASC (control), 0 μM ASC (depleted).	ASC-supplemented cells responded with a reduced transport of ASC and a coherent reduced SVCT1 expression (mRNA and protein). Depleted cells displayed increased ASC transport and increased SVCT1 expression. No changes were found for SVCT2.
Primary rat astrocytes [131]	Astrocytes were incubated with ASC (from 0 to 300 μM) in culture medium prior to measurements of uptake rates.	ASC depletion of culture medium increased the *V*_max_ by 15% after one hour and 20% after 6 h. ASC repletion resulted in a 20% decrease after one hour and 30% after 18 h.
Rat osteosarcoma cell line (ROS 17/2.8) [132]	Cells were incubated with ASC (from 0 to 300 μM) in culture medium prior to measurements of uptake rates.	ASC depletion of culture medium increased the *V*_max_ by 41% after six hours. ASC repletion resulted in a 40% decrease after six hours.
Porcine proximal tubule cell line (LLC-PK1) [128]	Cells were incubated with increasing concentrations of ASC in culture medium (10, 25, 50 and 100 μM).	Increasing concentrations in ASC reduced apical SVCT1 expression and induced translocation of SVCT1 to the cytoplasm before the signal was diminished.

Findings *in vitro* have shown a significant decrease in ASC uptake and concurrent mRNA expression-reduction following high-dose ASC culture, suggesting a putative feed-back mechanism and the association of transcriptional repressor elements in the regulation of ASC uptake [125,126] (Table 1). This has been further supported by studies in human hepatic cells, in which ASC depletion significantly increased SVCT1 expression [127]. The involvement of a transcriptional regulatory mechanism was confirmed by the finding of an altered promoter activity associated to the Hepatic Nuclear Factor 1 (HNF-1) binding sites on the SVCT1 promoter region [127]. The involvement of translational regulation has been shown for the SVCT2 transporter in human platelets [124]. A reduction of the ASC concentration in the surrounding medium to 30% increased the *V*_max_-value by almost 240%, whereas the *K*_m_-value remained unchanged, indicating a responsive increased transport rate [124]. This was later confirmed by a marked increase in SVCT2 protein expression compared to platelets prior to deprivation of ASC [124]. In renal proximal tubule cells, increasing concentrations of ASC led to a signal translocation from the apical membrane to the cytoplasm before the signal was diminished [128]. The effect was a 50% reduction in transport by cells pretreated with amounts of ASC corresponding to plasma saturation (50–100 μM), suggesting that apical levels play a pivotal role in the regulation of ASC uptake *in vitro*.

A substrate-dependent differential regulation has also been supported by *in vivo* studies (Table 2). Almost four decades ago, Rose and Nahrwold showed that guinea pigs receiving diets containing five or 25 times the vitC of a standard diet reduced the rate of ASC uptake by 32%–52% compared to control animals [129]. Daily intramuscular injections of 300 mg ASC reduced ASC influx into the intestinal mucosa by 16%, suggesting that high circulating ASC levels may inhibit transport across the intestinal epithelium, but not ruling out that ASC concentrations in the intestinal lumen or cytoplasm of epithelial cells contribute to the regulation [129]. In guinea pigs, a diet containing high amounts of vitC (5000 mg/kg feed) reduced ASC uptake across isolated ileac mucosa by 25%–50% in both adult male, lactating female and juvenile animals compared to controls (200 mg vitC/kg feed) [130]. The observed decrease in uptake did not result in alterations of *K*_m_-values, indicating that the reduced absorption rate was due to either reduced abundance or increased degradation of the transporting molecules [130]. However, guinea pig counterparts receiving low levels of vitC (3.5 mg/week) did not show an increased rate of ASC uptake in response to deficiency [130]. The authors suggested that transporters have a nearly complete extraction efficiency, even under standard conditions (control animals) [130]. Likewise, an absence of up-regulation of SVCT-transporters following long-term vitC deficiency (100 mg vitC/kg feed) in guinea pigs has been shown [31].

In humans, plasma concentrations following oral dosing of vitC are tightly regulated with peak plasma levels of around 200 μmol/L and a steady-state of 70–85 μmol/L, even when excessive amounts (3 g) vitC are ingested [25,133]. A steep decline in ASC bioavailability following increased oral doses suggests that intestinal transport is a key factor in maintaining whole body vitC homeostasis. This regulation may, however, be bypassed by intravenous dosage, achieving plasma concentrations well beyond the threshold of oral administration levels [26,133]. Concentrations in plasma have been shown to pose a direct effect on the tissue accumulation of ASC. Oral supplementation of mice unable to synthesize vitC due to a mutation in the l-gulono-γ-lactone gene (*gulo^−/−^*) has shown that increased plasma levels are required to achieve optimal concentration in several tissues (liver, heart and kidney) compared to levels necessary to obtain saturation in the brain [134]. This confirms the brain as being particularly efficient in retaining ASC, but also points towards a direct association between differences in plasma concentration and tissue-specific uptake [134]. In a recent intervention study in humans, the bioavailability of vitC in skeletal muscle relative to dietary intake was shown to correspond to plasma concentrations, whereas the same close correlation could not be found in leukocytes [135]. This could indicate an increased sensitivity in muscle towards alterations in plasma concentration or/and a differential uptake in leukocyte cells, possibly linked to differences in SVCT2 expression between the two cell types [135].

**Table 2:** Overview of *in vivo* studies regarding the regulation of vitC transport in response to vitC levels.

Animals species	Vitamin C regimen	Principal findings
Guinea pig [129]	ASC content in diet was increased by five- and 25-times compared to standard diets.	A reduction in ASC influx across the ileum by 32%–52% in animals fed high ASC diet compared to controls (standard).
Guinea pig [130]	Animals received either high (5000 mg/kg diet), low (0 mg/kg diet) or control (maintenance) (200 mg/kg diet) levels of vitC.	A high vitC level (hypervitaminosis) reduced the ASC rate of uptake across the intestinal brush border by 25%–50% compared to controls. Hypovitaminotic animals were not found to be different from controls.
Guinea pig [31]	Young and old animals, long-term on either control (325 mg vitC/kg) or deficient (100 mg vitC/kg) diets.	No effect of dietary vitC regimen on the expression of SVCT1 or SVCT2 mRNA in liver or brain.
Knockout mice (*smp30/gnl^−/−^*) [30]	Effects of vitC depletion *vs*. control (1.5 g vitC/L water) in wild-type (WT) and knockout (KO) mice.	In KO, mice vitC depletion increased SVCT1 and SVCT2 mRNA expression in the liver (by 21 and 55%, respectively) and increased SVCT1 by 55% in the small intestine compared to control counterparts. No changes were found in the kidney or cerebellum. In WT-mice, depletion increased SVCT2 expression in the small intestine by 43%.
Knockout mice (*gulo^−/−^*) [29]	*Gulo^−/−^* mice exposed to different ASC levels (drinking water): high 3.33 g/L; standard 0.33 g/L; low 0.033 g/L and depletion 0 g/L. WT mice were included as controls.	Depletion resulted in an increased mRNA expression of SVCT2 in the liver compared to WT controls. A trend towards increased protein levels of SVCT2 in liver and cerebellum was reported, although it did not reach a statistical level of significance.

An increase in SVCT-expression as a response to a reduction in ASC, followed by a decrease in SVCT mRNA when ASC levels increased, has been reported in developing teleost fish, which naturally are unable to synthesize vitC [136]. Although this could be part of a normal developmental sequence of events, it may also be a substrate-dependent response on SVCT-expression by which ASC uptake is regulated [136]. Studies of senescence marker protein-30 (SMP30)/gluconolactonase (GNL) knockout mice has revealed that vitC depletion increases SVCT1 mRNA levels in liver and small intestine and SVCT2 mRNA in liver [30]. VitC sufficient wild-type (WT) mice had 43% less SVCT2 mRNA compared to ASC-depleted WT mice, suggesting a deficiency associated mRNA upregulation. No changes were found in kidney or cerebellum of either WT or *smp30/gnl^−/−^* groups [30]. The study did not find any significant changes in the expression of GLUT-transporters (1, 3 and 4, respectively) [30]. In another study, Meredith *et al*. reported a significant increase in liver SVCT2 mRNA during vitC deficiency in the *gulo^−/−^* mice, whereas no effect was found in either cortex or cerebellum of the brain [29]. These findings may indicate the existence of an alternative transport mechanism involved in the regulation of vitC during deficiency [29]. In a study of fetal *vs*. maternal vitC status in guinea pigs, it was recently found that an inadequate maternal vitC intake during pregnancy resulted in reduced plasma vitC in newborn deficient pups, compared with their mothers [137]. The authors interpreted their findings as an indication that preferential transport of vitC from the mother to the fetus is overridden during a prolonged maternal vitC deficiency, thereby maintaining a basal maternal vitC concentration at the expense of the offspring [137]. Thus, several studies suggest that the exact mechanisms controlling the substrate-mediated regulation of vitC homeostasis in the body remains to be fully disclosed, but it appears likely that there are different mechanisms involved and that regulatory control may vary within specific tissues. Other regulatory mechanisms have also been suggested to contribute to vitC regulation, *i.e.*, feed-back sensing and hormonal regulation [26].

## 4. Concluding Remarks

VitC homeostasis is tightly regulated by a variety of more or less specific transport mechanisms, some of which may remain to be established. Several *in vitro* and *in vivo* studies have reported dose- and concentration-dependent rates of transport, both during ASC depletion and following supplementation, and these changes in transport activity apparently occur without change in affinity for the substrate. However, conflicting evidence has also been put forward in which the resulting changes in tissue compartment concentrations cannot be explained by alterations in the abundance of known vitC transporters. Moreover, as genetic variation has been shown to significantly influence vitC homeostasis *per se*, such information needs to be taken into consideration in future studies. Thus, continued efforts are required to establish the mechanisms by which the body efficiently adapts to declining vitC intakes.
